# Morpho‐Anatomical and HPTLC Investigations of *Lysimachia nummularia* L. (Primulaceae) Grown in Switzerland

**DOI:** 10.1002/jemt.70153

**Published:** 2026-05-07

**Authors:** Carolina Sabedotti, João Vitor da Costa Batista, Bettina Leonhard, Konrad Urech, Mahsa Ahmadinezhad, Jakob Maier, Stephan Baumgartner, Fabio Boylan, Carla Holandino, Jane Manfron

**Affiliations:** ^1^ Postgraduate Program in Pharmaceutical Sciences State University of Ponta Grossa Ponta Grossa Brazil; ^2^ Society for Cancer Research, Hiscia Institute Arlesheim Switzerland; ^3^ Department of Pharmaceutical Sciences, Division of Pharmaceutical Technology University of Basel Basel Switzerland; ^4^ Institute of Integrative Medicine, University of Witten/Herdecke Herdecke Germany; ^5^ Institute of Complementary and Integrative Medicine, University of Bern Bern Switzerland; ^6^ School of Pharmacy and Pharmaceutical Sciences, Trinity Biomedical Sciences Institute, Trinity Natural Products Research Centre, Trinity College Dublin Dublin Ireland; ^7^ Laboratório Multidisciplinar de Ciências Farmacêuticas, Faculdade de Farmácia Universidade Federal do Rio de Janeiro Rio de Janeiro Brazil

**Keywords:** HPTLC, *Lysimachia nummularia*, optical microscopy, scanning electron microscopy

## Abstract

Primulaceae are distributed all over the world, encompassing around 2590 species. The genus *Lysimachia* is also part of this family. *Lysimachia nummularia* L., popularly known as moneywort or creeping Jenny, has been used in Europe to treat many diseases. However, there are no reports of a complete morpho‐anatomical description for this species. The aim of this study was to characterize the morpho‐anatomical features of the plant, as well as to establish High‐Performance Thin‐Layer Chromatography (HPTLC)‐fingerprints of extracts prepared from the plant by decoction and infusion as well as the organic fractions obtained from these extracts. Standard techniques of light and scanning electron microscopy, as well as histochemical tests, were used; a standard method of HPTLC was applied. The species shows simple, opposite, membranaceous leaves, with rounded roots containing a central medulla and a ribbed stem with endoderm. Amphistomatic leaves bear glandular trichomes on the abaxial surface, also found on the petiole, midrib, petal, and filament. Histochemical analyses confirmed the presence of lipophilic compounds, proteins, starch, phenolics, tannins, and lignin. HPTLC analysis showed some differences in the fingerprints of the organic fractions examined, yet it highlighted the need for further studies regarding the chemical composition of the plant.

## Highlights

-
*Lysimachia nummularia* L. is known since it is used in European medicine at least since the 18th.
- There was no anatomical description for this plant.
- The morpho‐anatomical description of this study aids in the species identification.

## Graphical abstract

This research provides a complete morphoanatomical description of *Lysimachia nummularia* , as well as a HPTLC profile. Standard techniques of light and scanning electron microscopies, as well as standard techniques of HPTLC, were used.

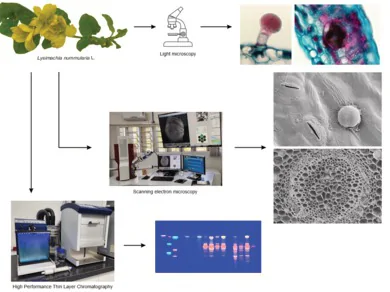

## 1 Introduction

The family Primulaceae Batsch ex Borkhausen (1797) belongs to the order Ericales and comprises the subfamilies Maesoideae, Theophrastoideae, Myrsinoideae, and Primuloideae, which were previously treated as different families. It is widely distributed around the world, covering 58 genera and about 2590 species (Stevens 2017). The subfamily Myrsinoideae Burnett is distributed in all types of tropical and temperate climate of the North Hemisphere, and was previously classified as a family, encompassing 33 genera and about 1250 species (Jung‐Mendaçolli 2005).

The genus *Lysimachia* Tourn. ex L., first described in 1753, comprises 288 species around the world (POWO 2025). Most species occur in the Northern Hemisphere, in temperate and subtropical climates, and may also reach the tropics, with several representatives in Asia, Africa, and South America (Linnaei 1753; Hu 1994; Hu and Kelso 1996; Hao et al. 2004). It is one of the largest genera of the Primulaceae family (Cronquist 1981; Takhtajan 1997). Due to a new assessment and delimitation, the tribe Lysimachieae was transferred to the subfamily Myrsinaceae (Anderberg and Stahl 1995; Anderberg et al. 1998; Källersjö et al. 2000).

*Lysimachia nummularia* L., popularly known as moneywort or creeping Jenny, is a creeping, low‐growing plant that covers the soil. This plant, being native to Europe, was naturalized in the Americas and nowadays is considered invasive in parts of eastern and northwestern areas of North America (Missouri Botanical Garden 2024). *Lysimachia nummularia* has been listed in the Codex Medicamentarius sive Pharmacopoea Gallica (Imbesi 1964) and has been used in Europe externally for treating wounds and ulcers and internally for diarrhea, salivation, gout, rheumatism, and joint pain (List and Horhammer 1976; Stehlin et al. 2024). Antioxidant, antimicrobial, and cytotoxic activities for its extracts have been reported. According to the literature, antioxidant activity can be attributed to phenolic compounds, especially flavonoid glycosides and triterpene saponins. Phenolic acids, benzoquinones, tannins, as well as other phenolic compounds have also been detected (Korta 1970; Yasukawa et al. 1990; Marr et al. 1998; Podolak and Strzałka 2008; Tóth et al. 2012, 2014; Podolak, Koczurkiewicz, Galanty, and Michalik 2013; Podolak, Koczurkiewicz, Michalik, et al. 2013; Deng et al. 2015; Hanganu et al. 2016; Özgen et al. 2020; Weissenstein 2021; Suçiu, Stoicescu, Lupu, et al. 2023a, 2023b). Although secretory cavities in its leaf blade have been described (Solereder 1908; Lersten 1986), there are no reports of a complete morpho‐anatomical description for this species.

Although there have been some studies regarding the phytochemical composition of different extracts from this species, so far, there is no investigation on a complete morpho‐anatomical description. Therefore, this study aimed to provide a detailed morpho‐anatomical description, as well as an HPTLC profile of *Lysimachia nummularia* infusions and their organic fractions.

## 2 Materials and Methods

### 2.1 Botanic Material

The botanical materials of *Lysimachia nummularia* L.—leaves, stems, flowers, and roots – were collected in Nuglar, Solothurn, Switzerland (47°28′16.9″ N 7°41′33.8″ E) in the morning of the 10th of June 2024, by B.L. and J.V.C.B. A voucher was prepared to compare to the one kept at the University of Basel's Herbarium (Switzerland), under the registration number of BASBG‐00090509.

### 2.2 Light Microscopy

After the harvest, a part of the plant material was separated for microscopic analysis. The organs of *L. nummularia* were fixed in ethanol: acetic acid: formaldehyde 70—FAA 70 (in a ratio of 5:5:90 v/v/v) for three days (Johansen 1940), then washed in distilled water and stored in ethanol 70% (v/v) (Berlyn et al. 1976).

The plant organs were sectioned transversely by freehand with the aid of a razor blade, and then submitted to double staining, exposing the sections to Astra blue and basic fuchsin (Kraus et al. 1998). The stained sections were arranged on a slide; a drop of 50% glycerin solution was added, then a coverslip was placed, and the edges were sealed with transparent nail polish (Berlyn et al. 1976).

For epidermal analysis of the leaf blade, sections of the adaxial and abaxial surfaces of 1 cm^2^ were exposed to a 50% hypochlorite solution until they appeared translucent. These sections were then washed three times in distilled water and neutralized with 5% acetic acid, followed by six additional washes in distilled water. Afterwards, the sections were stained with safranin (Fuchs 1963) for 1 min and then arranged as described in the previous paragraph.

### 2.3 Histochemical Analysis

To evaluate the chemical composition of products from cell metabolism and cell walls, the fresh plant organs were sectioned transversely. Then, sections were treated with the following reagents and dyes: NADI, which shows terpenes, with essential oils staining blue and oil resins staining red (David and Carde 1964); red oil, in which lipophilic compounds stain red (Jayabalan and Shah 1986; Pearse 1968); Sudan Black B and Sudan III, which show lipids in shades of dark blue to black and orange, respectively (Pearse 1972); Nile blue sulfate, in which neutral lipids stain pink and acidic lipids in blue (Cain 1947); Coomassie brilliant blue, which shows protein bodies that stain light blue (Fisher 1968); Xilidine Ponceau (XP), in which protein bodies stain red (Vidal 1970); Schiff's periodic acid, which stains neutral polysaccharides in magenta (O'Brien and McCully 1981); a solution of iodine 2%, which shows starch grains by staining them brown, purple, or black (Johansen 1940); a 0.002% ruthenium red solution, which stains pectins from red to pink (Johansen 1940); a 5% ferric chloride solution, which highlights phenolic compounds by staining them from brown to black (Johansen 1940); a 10% potassium dichromate solution, which shows total phenolic compounds in a reddish‐brown color (Gabe 1968); acid phloroglucinol, which stains lignins in pink (Johansen 1940); a 0.5% hydrochloric vanillin solution, which highlights condensed tannins, staining them red (Mace and Howell 1974); and the reagents of Dragendorff (Svendsen and Verpoorte 1983), Ellram and Wagner (Furr and Mahlberg 1981), which show alkaloids in shades of orange to reddish‐brown.

### 2.4 Microscale Measurements and Photographic Documentation

To calculate the Stomatal Index (SI), 20 random fields of leaf epidermis were photographed. Then the stomata were counted as *S* and the epidermal cells as *E*, later applying the following formula, described in the Brazilian Pharmacopeia (BRASIL 2024):

$$\mathrm{SI}=\frac{S}{E+S}*100$$

After obtaining 20 SI for the 20 fields photographed, the mean and standard deviation were calculated. The length and width of 20 randomly selected stomata from different fields were also measured to determine the average stomatal size.

The photographic records were taken using an Olympus Microscope CX41 with Phase Contrast and Darkfield (Japan), equipped with a UCMOS05100KPA camera (China).

### 2.5 Light Scanning Electron Microscopy

#### 2.5.1 Procedure 1

For ultrastructural analysis under a scanning electron microscope (SEM) performed at the University of Basel (Nano Imaging Lab facilities, Basel University, Swiss Nanoscience Institute, Switzerland), fresh samples of leaves, stems, and roots were used. The materials were fixed with a 4% glutaraldehyde solution for approximately 18 h, and then the samples were dehydrated in an increasing 80, 90, and 100% ethanolic series (Souza 1998); after that, they were dried at a critical point using the Tousimis Autosamdri 815 (Lewis Ave, Rockville, MD 20851, USA) and then metallized with gold using a Leica EM ACE600 equipment (Leica Microsystems, Wetzlar, Germany). Electromicrographs were recorded using a Zeiss GeminiSEM 450 scanning electron microscope (ZEISS Group, Oberkochen, Germany).

#### 2.5.2 Procedure 2

For SEM analysis carried out at the State University of Ponta Grossa, flowers of *L. nummularia* stored in 70% ethanol were dehydrated in an increasing ethanolic series (Souza 1998) and then dried at a critical point in a Balzers CPD 030 (BAL‐TEC AG, Balzers, Liechtenstein) garnished with liquid CO_2_ and later metallized with gold using Quorum equipment (model SC7620). Electromicrographs were recorded using SEM Mira 3 (Tescan, Brno‐Kohoutovice, Czech Republic).

### 2.6 Collection for the Infusions and Ethanolic Decoction

The plant was harvested in 2 days (10.6. and 17.6.2024), in the morning, with temperatures between 16°C and 20°C within 2 to 3 h each day. The harvested plants were collected in plastic bags and kept under cold and humid conditions in a Styrofoam box. Then the plant was washed under flowing tap water and air‐dried at room temperature. The unpleasant or dead leaves were removed. After that, the material was packed in plastic bags, weighed, and stored at 4°C until the pharmaceutical process (decoction) could start on the next day. To get the sorted fractions of leaves, blossoms, flower buds and roots, these parts were pinched off by hand, packed in plastic bags, weighed, and frozen directly at −20°C.

### 2.7 Ethanolic Decoction [(DER 1:3, Ethanol 30% (w/w)]

For the preparation of the ethanolic decoction, the fresh flowering above‐ground parts of *Lysimachia nummularia* L. harvested on June 10th, 2024, were used.

The *L. nummularia* ethanolic decoction was obtained from Society for Cancer Research (batch 2024‐06‐11) and prepared with a drug extract ratio from fresh plant to extract DER 1:3. Starting with 800 g of the comminuted fresh plant material the final amount of extract was adjusted to 2′400 g. Ethanol 30% (w/w) was used as the main extraction solvent. Prior to extraction also the loss on drying of the fresh plant material was determined with a Mettler Toledo Moisture Analyzer HB43 (Greifensee, Switzerland). The measured water content of 82.1% was considered for calculating the necessary amount of ethanol 96% (V/V) to achieve an extract with the final ethanol content of the 30% (w/w). The decoction process was performed under reflux during 30 min., adapted from the European Pharmacopeia (2025), method 1.2.8. After filtration, the decoction was stored under light protection in brown glass bottles at room temperature (15°C–25°C) until it was used to obtain organic fractions, a process described in item II.i.

### 2.8 Aqueous Infusions Preparation

The leaves, flowers and flower buds respectively infusions were prepared using the material stored at −20°C. To 100 g of the leaves and flowers, 500 mL of boiling water was added and allowed to cool for 30 min. For the flower buds preparation, 50 g were added to 250 mL of boiling water for 30 min, until left to cool. Then all infusions were filtered. After that, the prepared infusions were stored in glass bottles for further processing to obtain organic fractions, as described in item 2.i.

### 2.9 Organic Fractions Obtention

To obtain the organic fractions of the hydroethanolic decoction, the 2390 mL were subjected to concentration, with the aid of a rotary evaporator (Rotavapor RE 111, Büchi, Flawil, Switzerland), until the volume was reduced to 250 mL. After that, 250 mL of distilled water was added, thus obtaining a final volume of 500 mL.

Then, to the 500 mL of the infusions of the leaves, flowers and the decoction of the flowering above‐ground parts, in separation funnels, 10 × 100 mL of dichloromethane (ROTH, ROTHPURAN > 99%) was added (total volume of 1000 mL), and to the 250 mL of the infusion of the flower buds, 10 × 50 mL of dichloromethane were also added (total volume of 500 mL). Each aliquot of dichloromethane was added and mixed gently. After the separation of the phases, the lower phase (organic phase) was collected; the same process was repeated until the dichloromethane extracts for each part of the plant were obtained. The extracts were then stored in glass bottles for further concentration, at 38°C–42°C (Water Bath 461, Büchi, Flawil, Switzerland) under vacuum (Vacuum Pump V‐700, Büchi, Flawil, Switzerland).

The same process described above was repeated with ethyl acetate (ROTH, ROTISOLV HPLC) and *n*‐butanol (Roth, ROTIPURAN ≥ 99.4%), to obtain fractions extracted with ethyl acetate and *n*‐butanol; however, in the case of these last two solvents, the fraction collected from the separation funnel was the higher (organic phase).

### 2.10 HPTLC—High‐Performance Thin‐Layer Chromatography

#### 2.10.1 Sample Preparation

Table 1 presents all the fractions that went through the solubilization process, the solvents used, and the volume of solvents used to solubilize the samples. For the dichloromethane fraction, dichloromethane was used combined with methanol (1:1), taking 1 mL of each solvent, so the final volume would consist of 2 mL. After the preparation of the samples, it was observed that the butanol fraction of the leaves and the butanol fraction of the ethanol decoction were more concentrated; they were diluted with methanol in a proportion of 2:8 (fraction: solvent).

**TABLE 1:** Fractions, solvents and volume used in the preparation of the samples for HPTLC.

	Fraction	Solvent(s) used	Amount (mL) of solvent used
1	Infusion of leaves—dichloromethane	Dichloromethane: Methanol (1:1)	2
2	Infusion of blossoms—dichloromethane	Dichloromethane: Methanol (1:1)	2
3	Infusion of flower buds—dichloromethane	Dichloromethane: Methanol (1:1)	2
4	Ethanol decoction—dichloromethane	Dichloromethane: Methanol (1:1)	2
5	Infusion of leaves—ethyl ester acetate acid	Methanol	2
6	Infusion of blossoms – ethyl ester acetate acid	Methanol	2
7	Infusion of flower buds—ethyl ester acetate acid	Methanol	2
8	Ethanol decoction—ethyl ester acetate acid	Methanol	2
9	Infusion of leaves—*n*‐butanol	Methanol	2
10	Infusion of blossoms—*n*‐butanol	Methanol	2
11	Infusion of flower buds—*n*‐butanol	Methanol	2
12	Ethanol decoction—*n*‐butanol	Methanol	2

#### 2.10.2 Reference Compounds Preparation

The reference compounds for the flavonoids analysis quercetin dihydrate, rutoside trihydrate, and myricetin were prepared at a concentration of 1 mg/mL in Ethanol 96%, whereas chlorogenic acid, hyperoside, and caffeic acid were prepared at a concentration of 0.5 mg/mL in methanol. For the saponins analysis, the reference compounds Saponin and Saponin Quillaja were prepared at a concentration of 5 mg/0.5 mL in methanol. A raw ethanolic decoction prepared in the previous year (batch 2023‐06‐27) was also used as control.

#### 2.10.3 Instrumentation

A HPTLC system (CAMAG, Muttenz, Switzerland), equipped with Automatic TLC Sampler 4, Automatic Developing Chamber 2 (ADC2, CAMAG), TLC Scanner 3 (CAMAG), TLC Plate Heater III (CAMAG), Derivatizer (CAMAG) (Automated spraying device for reagent transfer onto TLC/HPTLC plates) and a Reprostar (CAMAG) for illumination together with a Lumix digital camera DMC‐TZ61 (Panasonic, Osaka, Japan) was used to conduct the HPTLC analysis. Analyses were run and evaluated using the winCATS Planar Chromatography Manager software version 1.4.6. (CAMAG). HPTLC parameters in accordance with the European Pharmacopeia (2025), method 2.8.25.

#### 2.10.4 HPTLC Analysis

Prior to the analysis itself, each one of the HPTLC silica gel 60 F245 plates (Supelco, Merck, Darmstadt, Germany) used was predeveloped with methanol and chloroform (1:1), then dried at 120°C for 20 min on the plate heater.

The 2 μL of the samples and reference compound solutions were applied as narrow bands, 8 mm in length, at a distance of 8 mm from the lower edge of HPTLC plate, previously predeveloped, then developed with ethyl acetate, water, formic acid, and glacial acetic acid (100:26:11:11) (v/v) (Reich and Schibli 2007). The migration distance was 70 mm. The detection was performed under UV at 254 nm, UV at 366 nm and white light in reflection + transmission (RT) prior to derivatization and under UV at 366 nm and white light (RT) after derivatization with natural product/polyethylene glycol reagent (NP/PEG) (Reich and Schibli 2007).

#### 2.10.5 Densiometric Analysis

The tracks were scanned at 280 nm using a TLC Scanner 3 set in adsorption mode starting on 5.0 mm until 75.0 mm with a scanning speed of 20 mm/s; the slit was set at 4.00 × 0.30 mm and data resolution at 100 μm/step. The densitograms in UV‐ resp. VIS‐light of the zones were recorded at 366 nm for the screening of flavonoids, respectively at 530 nm for the saponins.

#### 2.10.6 Derivatization Reagents Preparation and Spraying

Natural product reagent (NP): 1 g of diphenylborinic acid aminoethyl ester was dissolved in 200 mL of ethyl acetate.

Polyethylene glycol reagent (PEG): 10 g of polyethylene glycol 400 was dissolved in mL of dichloromethane. 200.

The derivatization reagents were sprayed using an immersion device III with a green nozzle, spraying 2 mL level 4 for NP and a blue nozzle for PEG reagent. Prior to derivatization with NP reagent, the plate was heated at 110°C for 3 min.

#### 2.10.7 Retention Factor (*R*_f_) calculus

To calculate the retention factor (*R*_f_), the distance traveled by the compound was divided by the distance traveled by the mobile phase, in which both were measured from the origin of application, in cm (BRASIL 2024).

## 3 Results and Discussion

To the best of our knowledge, this is the first paper describing detailed morpho‐anatomical features of the whole plant *Lysimachia nummularia* L.

### 3.1 Morphology

**FIGURE 1:**
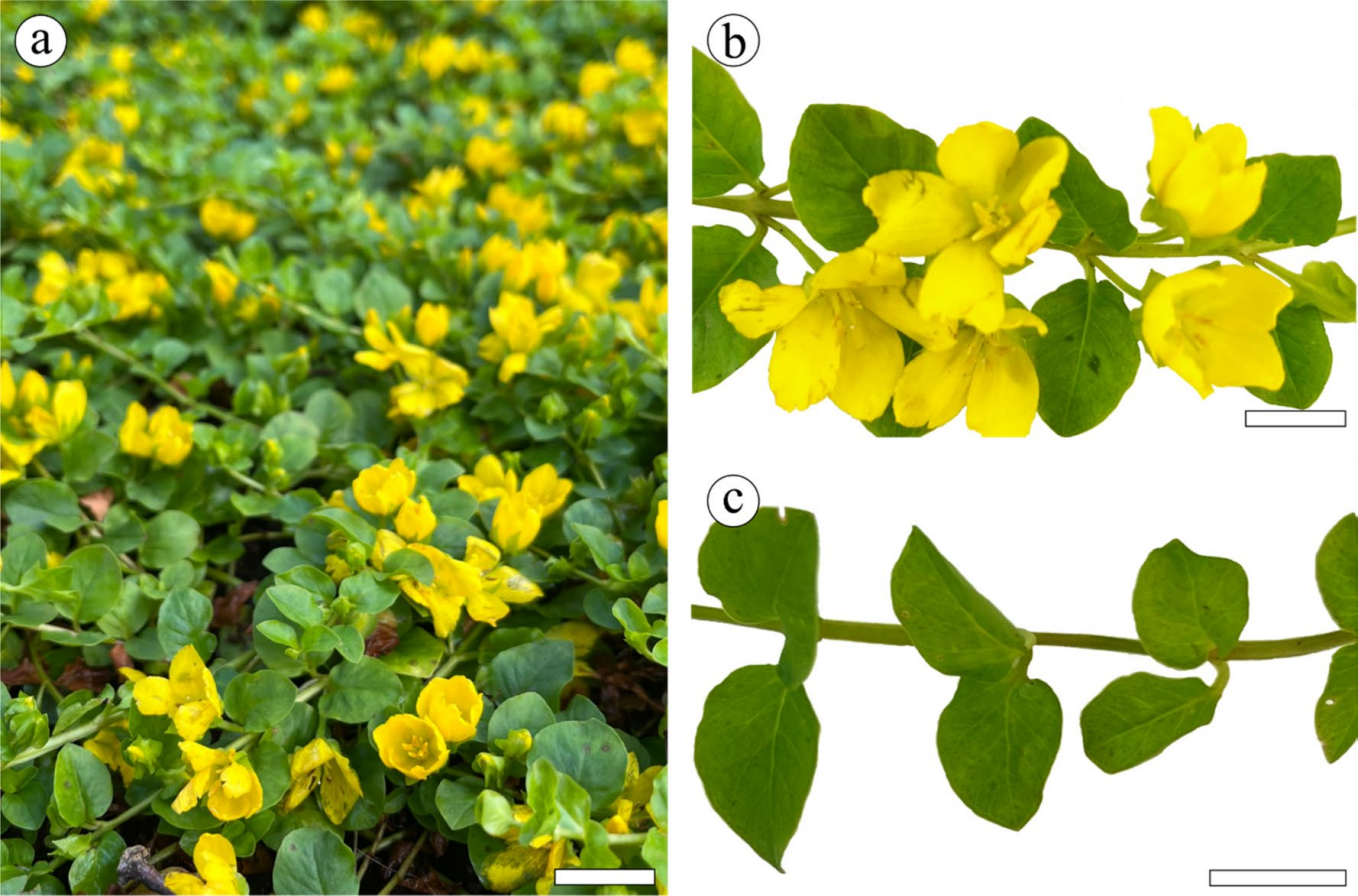
Morphology of *Lysimachia nummularia* . Note: (a) Plant in habitat. (b, c) Twig with leaves and flowers of *L. nummularia* . Scale bar: (a) 2 cm; (b, c) 1 cm.

*Lysimachia nummularia* is an herbaceous perennial, low‐growing, ground covering creeping plant (Figure 1a), more thriving in moist soils. The leaves are simple, opposite, membranaceous and oval to rounded in shape, with acute to obtuse apex and reentrant base, entire margins, pinnate veins, green leaf colored (Figure 1b,c). Shape and size can vary from oval to cordiform, measuring 1.90 ± 0.19 cm in length and 1.60 ± 0.24 cm in width. The average petiole was 0.39 ± 0.07 cm.

Single flowers (Figure 1b) can be found linked to the stem by a simple pedicel to a regular, dialissepal and campanulated chalice, formed by five small greenish sepals. The regular, dialipetal and rosacea receptacle has five petals yellow colored; they present acute to obtuse apex and velvety consistency. Stamen (Figure 5g), in a number of five, are connected to each other in adelphy. Anthers (Figure 5g) are sagittarian and valved, with the apex more tapered than the base. The pistil is provided with a globulous ovary; the style is terminal with a penicillated stigma.

These findings agree with the reported data for *L. nummularia* 's aerial parts (Linnaei 1753; Small 1933).

### 3.2 Anatomy

**FIGURE 2:**
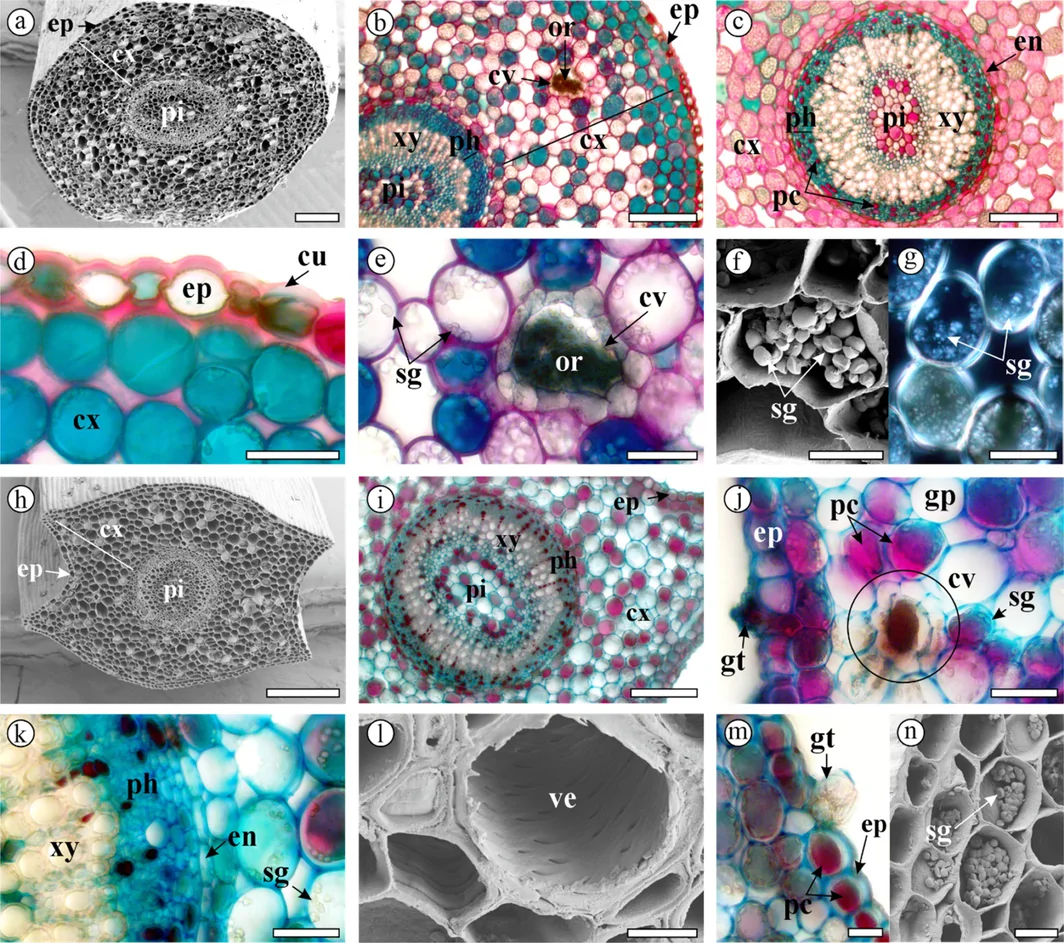
Anatomy of *Lysimachia nummularia* 's roots and stems. Note: Roots (b–g) and (h–n) stems in (b–e, i–k, m) light microscopy, (a, f, h, l, n) scanning electron microscopy and (g) polarized light. (cv, secretory cavity; cu, cuticle; cx, cortex; en, endodermis; ep, epidermis; gt, glandular trichome; pc, phenolic compounds; ph, phloem; pi, pith; sg, starch grains; ve, vessel element; xy, xylem). Scale bar: (h) 300 μm; (a–c, i) 200 μm; (d, e, g, j, k) 50 μm; (m) 25 μm; (f, n) 20 μm; (l) 10 μm.

*Lysimachia nummularia* 's secondary structure roots are rounded in shape (Figure 2a). The root has a one‐layered epidermis (Figure 2b,d) covered by a slightly thick cuticule (Figure 2d). Below the epidermis, in the cortical parenchyma, starch grains (Figure 2e–g) are found in almost every cell. Also present in the cortex, there are secretory cavities filled with lipophilic content (Figure 2b,e). An endodermis is present between the cortex and the collateral vascular system, having the phloem facing the endodermis and the xylem facing the center (Figure 2c). In the center of the root, a pith can be found (Figure 2c); the cells in the pith contain starch grains.

The root's endodermis is uniseriate and has typical Caspary striations, controlling the flow of substances to the central cylinder, a common feature of plant roots. As will be reported later, the endodermis persists on the stem, which is not uncommon for eudicots (Metcalfe and Chalk 1950).

The presence of central pith in roots is not common. However, there are exceptions in some herbaceous eudicots: certain roots have a siphonosthelic organization, with a polyarchic vascular cylinder and parenchymal tissue in the center. In these medullary roots, the center is occupied by medullary parenchyma, often with aeration spaces (aerenchyma). *L. nummularia* grows in moist habitats. Due to its habit and environment, its roots are expected to exhibit anatomical adaptations similar to other species of the genus, with a pith present. An example of pith present in a *Lysimachia* species' root was reported by Bejenaru et al. (2013), which described for *Lysimachia punctata* L. a polyarchic internal organization, with pith tissue present in the central region of the root, the authors described this central parenchyma as meatous, suggesting an aerenchyma (Metcalfe and Chalk 1950; Bejenaru et al. 2013). In the present study, *L. nummularia* was not collected in a moist soil or near any water body; thus, the amilyferous parenchyma found in the root's pith could be replacing the aerenchyma that can be present in *Lysimachia* species, due to the environmental change.

The stem is a four‐rib rounded shape (Figure 2h) and primary structure. The epidermis is unilayered, covered by a thin cuticle (Figure 2j,m); capitated glandular trichomes are present (Figure 2m). Phenolic compounds are found in the epidermal cells (Figure 2m), as well as in the cortical parenchyma (Figure 2j). Cells containing starch grains (Figure 2j,k,n) and lipophilic secretory cavities are also present in the cortical parenchyma (Figure 2j). The cortical parenchyma is separated from the vascular system by an endodermis (Figure 2k). The collateral vascular system has phloem toward the periphery, and xylem facing the pith (Figure 2i); the xylem is pitted (Figure 2l), according to Ray (1972). Phenolic compound‐producing cells are also found between the phloem, parenchymatic rays and in the pith (Figure 2i,k). Starch idioblasts are also present in the pith.

Bejenaru et al. (2013), when describing the secondary structure stem of *L. punctata* , reported pluricellular uniseriate and/or bicellular non‐glandular trichomes and capitated glandular trichomes on the stem's surface. In the present study, *L. nummularia* presents only glandular trichomes.

**FIGURE 3:**
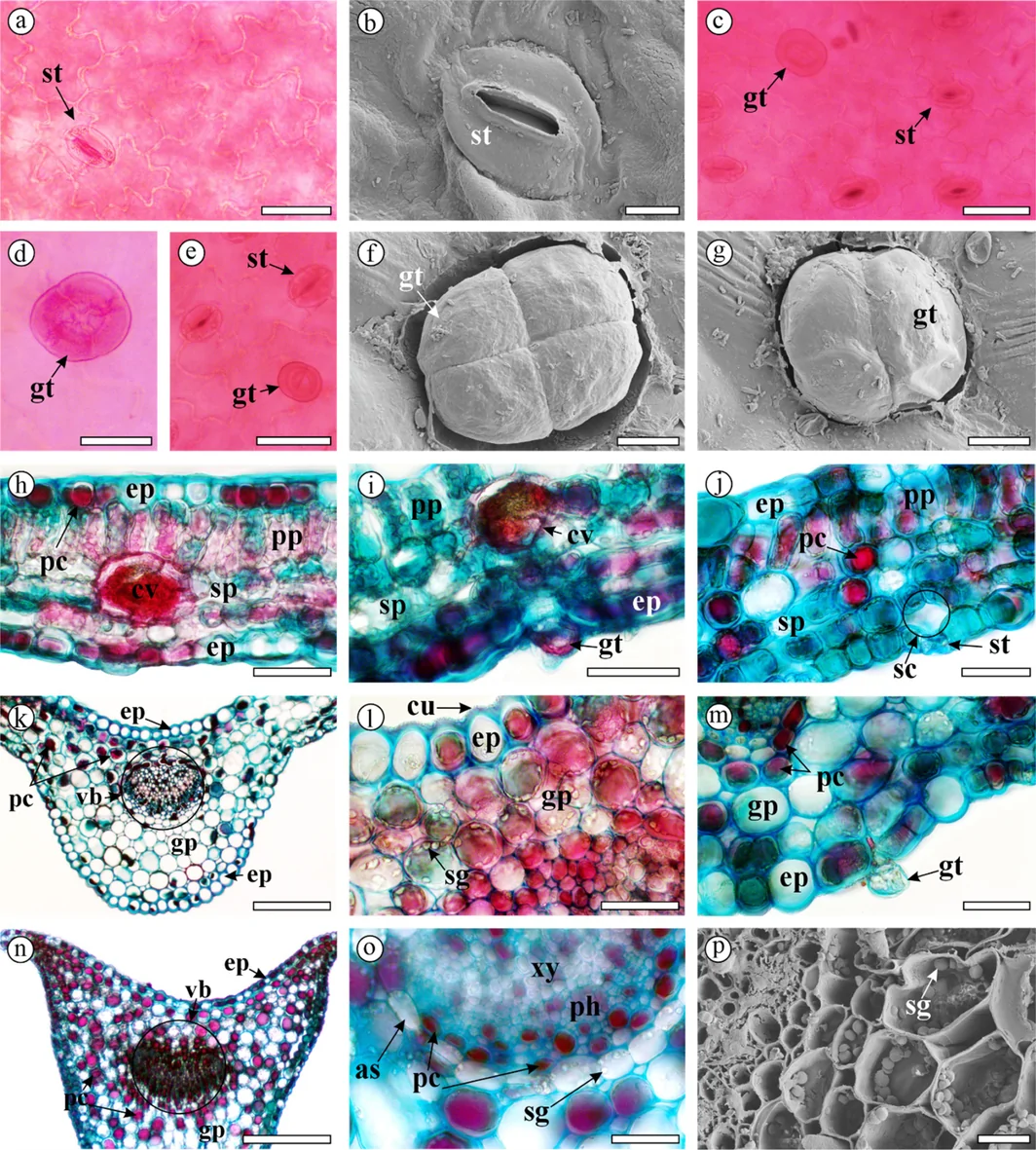
Anatomy of *Lysimachia nummularia* 's leaves. Note: Leaves' (a, b) adaxial and (c–g) abaxial surfaces, (h–j) mesophyll, (k–m) midrib and (n–p) petiole in (a, c–e, h–o) light microscopy and (b, f, g, p) scanning electron microscopy (cv, secretory cavity; cu, cuticule; epidermis; gp, ground parenchyma; gt, glandular trichome; pc, phenolic compounds; ph, phloem; pp., palisade parenchyma; sc, substomatal cavity; sg, starch grain; sp., spongy parenchyma; st, stomata; vb, vascular bundle; xy, xylem). Scale bar: (k, n) 200 μm; (a, c, e, h–j, l, m, o) 50 μm; (d) 25 μm; (f, g, p) 10 μm; (b) 8 μm.

On surface view, both adaxial (Figure 3a) and abaxial (Figure 3c) epidermis presented thin and sinuous anticlinal cell walls, covered by a thin cuticle. Anomocytic stomata (Figure 3a–c,e) can be seen at the same level as the epidermic cells on both sides, thus, the leaves can be classified as amphistomatic; although, there are more stomata on the abaxial side, as the calculated Stomatal Index (SI) pointed out, being 8.06% ± 3.01% and 16.89% ± 4.19% for the adaxial and abaxial surface, respectively. Howerver, there was no statistically significant difference in the average size of the stomata, as for the adaxial side they measured 31.32 ± 2.84 μm long and 21.65 ± 1.03 μm wide and for the abaxial side they measured 30.90 ± 1.90 μm long and 21.20 ± 2.02 μm wide.

Sinuous anticlinal cell walls and thin cuticle are common features of Myrsinoideae subfamily. Some species from genera *Ardisia* Sw., *Cybianthus* Mart., *Myrsine* L. and *Stylogyne* A.DC. reportedly present anisocytic or paracytic stomata and hypostomatic leaves (de Luna et al. 2017), which differ from *L. nummularia* , differentiating this species from other Myrsinoideae.

Despite the type of stomata, the percentage of SI (%) and the stomatal size can be affected by the environment (Liu et al. 2023), they have an important role in differentiation of *Baccharis* L. (Antunes et al. 2024) and *Eucalyptus* L'Hér (Migacz et al. 2018; de Brito et al. 2021) species, both known for their morphological similarity. Besides, Brazilian Pharmacopeia considers the SI as a useful parameter to the diagnosis and identification of the pharmacopeial plants (BRASIL 2024).

Capitate glandular trichomes can be seen only on the abaxial epidermis (Figure 3c–g), which differs from other Myrsinoideae species, whose trichomes are present on both leaves' surfaces (de Luna et al. 2017). Plus, the only species to have presented the same glandular trichome as *L. nummularia* is *Myrsine villosissima* Mart. (de Luna et al. 2017). The type, location, and presence of trichomes have a key role in the identification and differentiation of many morphologically similar species (Budel et al. 2018; de Almeida et al. 2021; Antunes et al. 2023).

**FIGURE 4:**
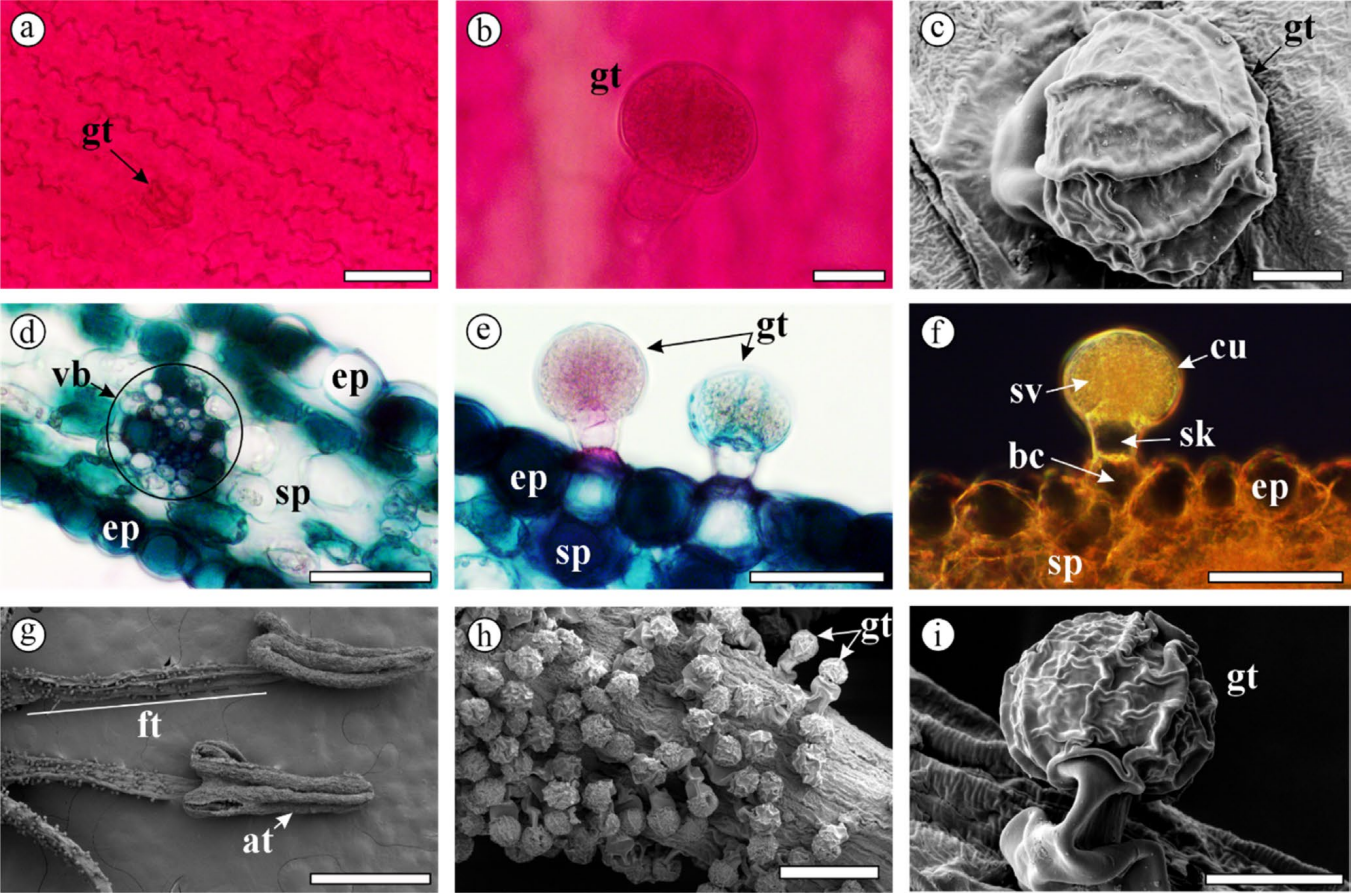
Anatomy of *Lysimachia nummularia* 's flowers. Note: Flower's (a–f) petals and (g–i) stamen in (a, b, d, e) light, (f) polarizing and (c, g–i) scanning electron microscopy (at, anther; bc, basal cell; cu, cuticle; ep, epidermis; ft., filament; gt, glandular trichome; sk, stalk; sp., spongy parenchyma; sv, storage cavity; vb, vascular bundle). Scale bar: (g) 1000 μm; (h) 100 μm; (a, d, e, f) 50 μm; (b) 25 μm; (i) 20 μm; (c) 10 μm.

In cross‐section, it can be seen a dorsiventral mesophyll composed of one layer of palisade parenchyma and 3–4 layers of spongy parenchyma (Figure 3h,j), covered by one layer of rounded epidermal cells on both sides of the lamina; these epidermal cells are covered by a thin cuticle. Small vascular bundles are in spongy parenchyma. Capitate glandular trichomes (Figure 3c–g) are seen on the abaxial side of the leaves; they are composed of one basal cell, one stalk cell, and the number of secretory cells and thus, storage cavities can vary from one up to four; the head of the capitate trichomes are covered by a thin cuticle (Figure 4f). Secretory cavities containing lipophilic material can be found in the mesophyll, especially between the palisade and the spongy parenchymas (Figure 3h,i). Phenolic compounds are found all over the mesophyll cells (Figure 3h,j), especially in the epidermis and in the spongy parenchyma cells.

Unilayered epidermis, dorsiventral mesophyll and secretory cavities are frequently reported for Myrsinoideae species (de Luna et al. 2013, 2017). *L. punctata* , however, did not present secretory cavities in the mesophyll or thin cuticle, and in contrast to *L. nummularia* , non‐glandular trichomes were reported on both sides of the leaves' lamina (Bejenaru et al. 2013); thus, despite having similar leaf structure, *L. nummularia* can be easily differentiated from *L. punctata* .

The midrib, in cross‐section, presents a concave‐convex shape (Figure 3k). The epidermises are unilayered, covered by a thin cuticule (Figure 3l); capitated glandular trichomes are also present in the midrib (Figure 3m). Straightly below the epidermises, a ground parenchyma (Figure 3k–m) is observed, in which cells containing phenolic content are detected. Starch idioblasts are also seen in the midrib (Figure 3l). The vascular system is collateral, in open arc shape and is involved by a bundle of phenolic content producing cells (Figure 3k). Phenolics are also present in the phloem.

Concave‐convex shaped midrib happens among other Myrsinoideae species, although it is not very common as flat‐convex or biconvex shape. Unilayered epidermis and collateral open arc vascular bundle are reported also for Myrsinoideae species; however, all species from genera *Ardisia*, *Cybianthus*, *Myrsine*, and *Stylogyne* present a sclerenchymatic sheath involving the vascular system (de Luna et al. 2013, 2017). *L. nummularia* , on the other hand, does not present this feature, which is another characteristic that helps to differentiate *L. nummularia* from the other Myrsinoideae species.

The petiole in the medial, in cross‐section, is concave‐convex with two lateral ribs (Figure 3n). Similarly, to the midrib, epidermises are unilayered, covered by a thin cuticule. In the ground parenchyma (Figure 3n) are found cells containing phenolic content and starch idioblasts (Figure 3o). The collateral vascular system is also in an open arc and is encircled by a sheath containing starch grains (Figure 3o). The same way as presented by the midrib: phenolic compound cells are present in the phloem (Figure 3o).

The reported shape for most of the petioles of Myrsinoideae species is flat‐convex. Brachysclereids are present in some species and the collateral open arc vascular bundle is mentioned to be present only in *Cybianthus venezuelanus* Mez, *Cybianthus verticillatus* (Vell.) G. Agostini, and *Stylogyne sordida* Mez; however, differently from the midrib, not all species reported presented a sclerenchymatic sheath involving the vascular system (de Luna et al. 2017); therefore, the concave–convex shaped petiole with two lateral ribs differentiates *L. nummularia* from the other Myrsinoideae species.

In addition, the shape, the open arc vascular bundle, the presence of phenolic compounds, and the lack of non‐glandular trichomes in both the midrib and petiole can help in the differentiation of *L. nummularia* and *L. punctata* , as the second also presents non‐glandular trichomes on both midrib and petiole, as well as an open arc with two dorsal traces as the vascular system in the petiole (Bejenaru et al. 2013).

A feature reported for almost all the Myrsinoideae species, which is the presence of calcium oxalate druses and/or prismatic crystals (de Luna et al. 2013, 2017), was not observed in any organ analyzed of *L. nummularia* . Calcium oxalate crystals are present in many plant families and play a key role in the differentiation of anatomically similar species; the presence, location and crystal morphotype can be used as anatomical markers (Franceschi and Nakata 2005; de Brito et al. 2021; de Almeida et al. 2021; Raeski et al. 2023; Antunes et al. 2024). Interestingly, Bejenaru et al. (2013) also did not report any occurrence of oxalate crystals in *L. punctata* 's organs; more studies on this topic are required to elucidate if the *Lysimachia* genus does not present calcium oxalate crystals or if the lack of crystals is due to the different environmental conditions in which the species were cultivated, such as Brazil, for Myrsinoideae species (de Luna et al. 2013, 2017); Romania for *L. punctata* (Bejenaru et al. 2013), and Switzerland for the *L. nummularia* harvested for the present study.

As for the flowers, the epidermis of petals also presents thin and sinuous anticlinal cell walls (Figure 4a), covered by a thin cuticle, in surface view. Although, differently from the leaves, the petals have capitate glandular trichomes on both sides of the epidermis (Figure 4b,c). These trichomes are also present in the filament of the anther (Figure 4h).

In cross‐section, the petals present an unilayered epidermis on both surfaces (Figure 4d). Beneath, a spongy parenchyma is found (Figure 4d,e); vascular bundles of small size can be found in this parenchyma (Figure 4d).

The capitate glandular trichomes, found in the stem, petiole, leaves' abaxial side, filament and both sides of petals, in cross‐section, are composed of a basal cell, stalk, secretory cells, and a storage cavity, as shown in Figure 4f,i, in polarized light microscopy and in scanning electron microscopy, respectively.

### 3.3 Histochemical Tests

**FIGURE 5:**
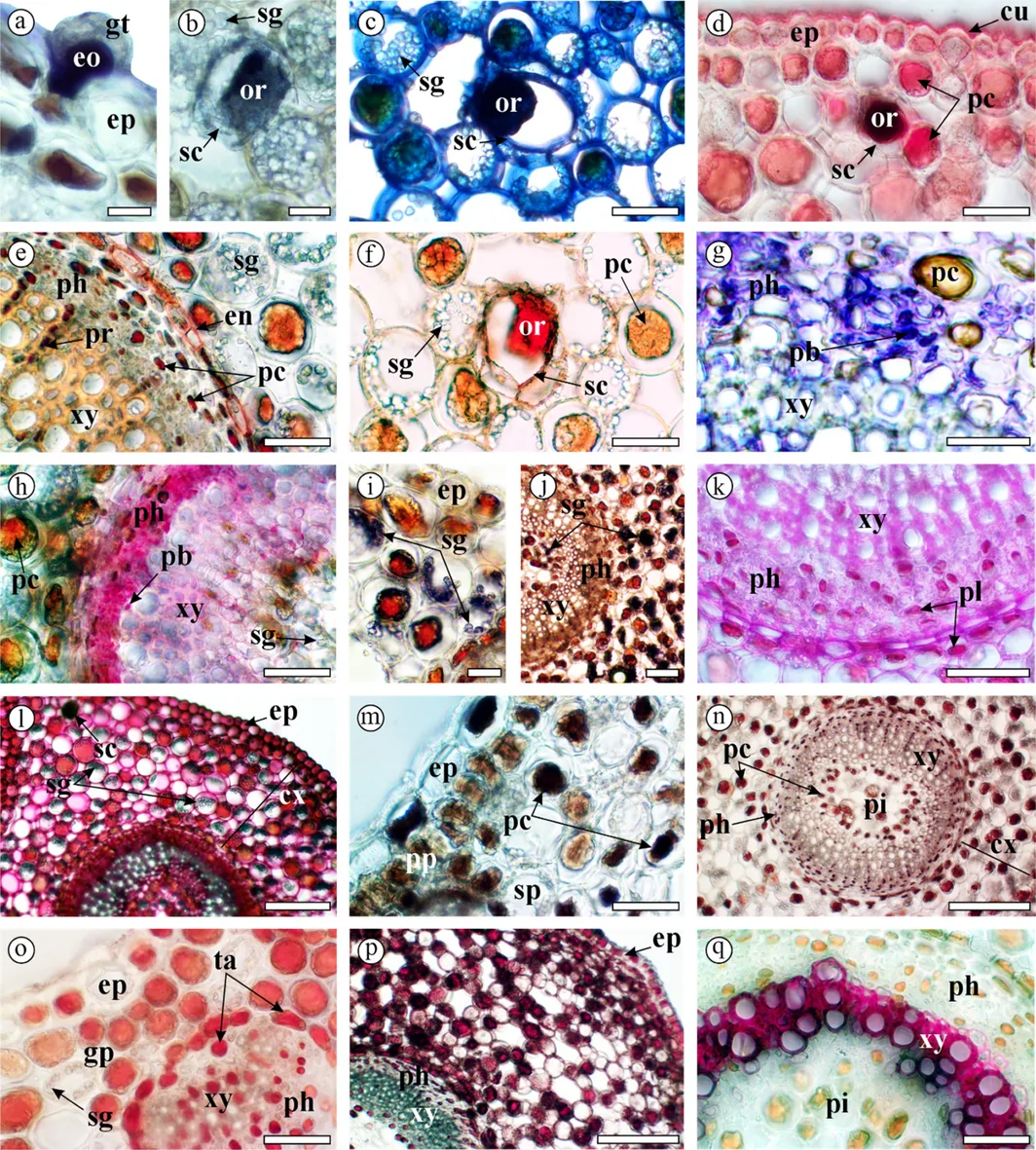
Histochemistry of *Lysimachia nummularia* in light microscopy. Note: (a) Petiole reacting with NADI. (b) Root after treatment with NADI reagent. (c) Root after reaction with Nile blue. (d) Stem stained with Oil Red. (e) Stem after exposure to Sudan III, highlighting the endodermis. (f) Stem after treatment with Sudan III, highlighting the secretory cavity. (g) Stem stained with Coomassie blue. (h) Stem after treatment with Xilidine Ponceau. (i) Mesophyll treated with iodine solution. (j) Stem after staining with iodine solution. (k) Stem reacting with Periodic Acid Schiff reaction. (l) Stem after staining with ruthenium red. (m) Mesophyll after reaction with ferric chloride. (n) Stem after exposure to potassium dichromate. (o) Midrib after treatment with vanillin/HCl. (p) Stem after staining with vanillin/HCl. (q) Stem after reaction with phloroglucin/HCl (ct, cuticle; cx, cortex; en, endodermis; eo, essential oil; ep, epidermis; gp, ground parenchyma; gt, glandular trichome; or, oil resin; pb, protein bodies; pc, phenolic compound; ph, phloem; pi, pith; pl., polysaccharide; pp., palisade parenchyma; pr, parenchymatic ray; sc, secretory cavity; sg, starch grains; sp., spongy parenchyma; ta, tannin; xy, xylem). Scale bar: (n, p) 250 μm; (l) 150 μm; (j) 100 μm; (c–f, h, k, m, o, q) 50 μm; (a, b, g, i) 25 μm.

All parts of *L. nummularia* analyzed reacted positively with NADI reagent, showing lipophilic compounds in cuticles of the leaves, petioles, stems, roots, and petals (Figure 5a) and in the endodermis present in the stem and roots; this reagent also reacted with the lipophilic content of the secretory cavities (Figure 5b), present in the mesophyll, petiole, stem, and root, staining these compounds in purple, which is indicative of essential oil and resin mixture, also called oil resins; the capitate trichomes contain storage cavities which were stained in blue color, suggesting the presence of essential oils (David and Carde 1964).

Nile blue sulfate (Figure 5c) stained in blue the secretory cells of all *L. nummularia* 's organs analyzed. This result highlights the presence of acid fats on the composition of the cuticule, the endodermis, the oil resins and the essential oils (Cain 1947). Oil red, Sudan Black B and Sudan III also react with lipophilic compounds, staining them in red, black and orange, respectively (Jayabalan and Shah 1986; Pearse 1972). The cuticle (Figure 5d), endodermis (Figure 5e), essential oils and oil resins (Figure 5d,f) all react with those three stains.

Coomassie Brilliant Blue stains proteins in light blue and Xilidine Ponceau stains them in red (Fisher 1968; Vidal 1970). Protein globular corpuscles are found on the phloem of all organs analyzed (Figure 5g,h).

Iodine solution detects starch grains staining them in dark purple to black (Johansen 1940). In *L. nummularia* , starch grains are found in the stems and roots' cortex and pith (Figure 5j); these grains were also found in midribs and petiole's ground parenchyma and mesophyll's spongy parenchyma (Figure 5i), which is untypical, since starch's function is energetic reserve (Taiz et al. 2024).

Periodic Acid Schiff (P.A.S.) reagent is also used to reveal polysaccharides, more specifically, neutral polysaccharides; hydroxyls on the polysaccharides are oxidized with periodic acid, forming aldehyde groups, which will bond with the colorless Schiff reagent, forming a magenta coloration (O'Brien and McCully 1981). *Lysimachia nummularia* tested positively with P.A.S., in the ground parenchyma midrib and petiole of the leaves and in the cortex, parenchymatic rays and pith of stems and roots (Figure 5k).

The diverse and complex group of gel forming polysaccharides is called pectin, which is the most abundant component of primary cell walls (Taiz et al. 2024). Ruthenium red is widely used to detect pectins, nucleic acids, and acidic mucilages in red (Johansen 1940). In the present study, all tissues from all *L. nummularia* 's organs analyzed reacted positively with this stain (Figure 5l), except for xylem, fibers, and starch grains.

Ferric chloride and potassium dichromate solution are used to detect phenolic compounds, producing a reddish brown and brown to black coloration, respectively, when the reaction is positive (Johansen 1940; Gabe 1968). Phenolic compounds are present in the epidermises, palisade and spongy parenchyma, cortical parenchyma, phloem and parenchymatic rays (Figure 5m,n).

Hydrochloric vanillin is used to detect tannins; it is based on the interaction of the aldehyde groups of vanillin with the phenolic groups present in the tannins, forming a red condensate product (Mace and Howell 1974). Tannins are present in all *L. nummularia* 's organs analyzed, especially in the epidermises, palisade and spongy parenchymas, cortical parenchymas, phloem and parenchymatic rays (Figure 5o,p).

To expose the presence of lignified elements, phloroglucinol/HCl is frequently used, staining these elements in pink (Johansen 1940). Lignin is a phenolic compound present in secondary wall structure, as for example, xylem, fibers, and sclereids (Taiz et al. 2024). In this study, lignin was found only in the xylem from vascular bundles, in all the organs analyzed (Figure 5q).

Dragendorff, Ellram and Wagner reagents are used to detect alkaloids, staining these compounds in orange to reddish brown color (Furr and Mahlberg 1981; Svendsen and Verpoorte 1983). None of *L. nummularia'*s parts analyzed reacted positively when exposed to any of these reagents.

### 3.4 High‐Performance Thin‐Layer Chromatography

**FIGURE 6:**
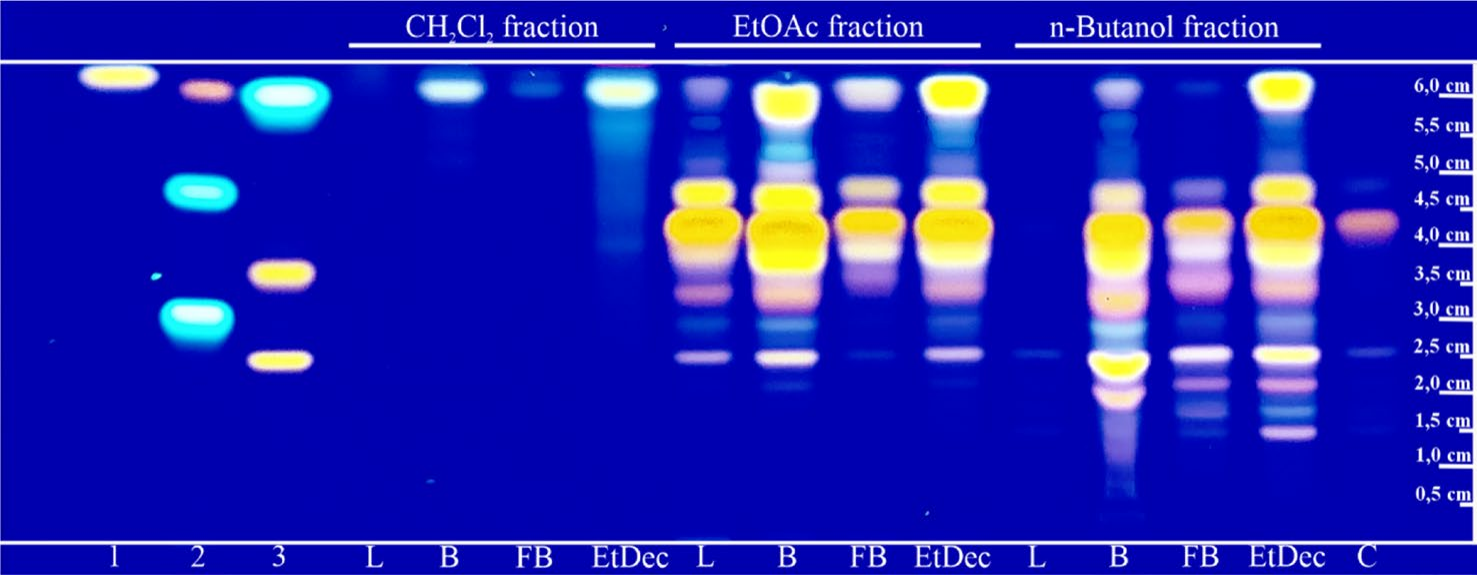
High‐performance thin‐layer chromatography (HPTLC) fingerprints of *Lysimachia nummularia* . Note: Dichloromethane (CH_2_Cl_2_), Ethyl Acetate (EtOAc), and *n*‐Butanol fractions of the infusions of leaves (L), blossoms (B), and flower buds (FB), and the ethanol decoction (EtDec) developed with mobile phase ethyl acetate, water, formic acid, and glacial acetic acid (100:26:11:11 v/v), detection at 366 nm after derivatization with natural product/polyethylene glycol reagent (NP/PEG). Standards: 1. quercetin 200 μg/mL; 2. chlorogenic acid 0.5 mg/mL; and myricetin 200 μg/mL; 3. rutoside 200 μg/mL, hyperoside 0.5 mg/mL, and caffeic acid 0.5 mg/mL; and (C) the raw ethanolic decoction from the previous year (batch 2023‐06‐27). Note: The white line at the bottom of the plate represents the start, and the white line in the upper part of the plate is the end of the development.

Figure 6 shows the high‐performance thin‐layer chromatography (HPTLC) fingerprints after the test for flavonoids in the best visualizing mode.

To detect the presence of flavonoids and phenolic acids in the infusions and ethanol decoction fractions, the following flavonoids were selected as standards: quercetin (*R*_f_ ~ 0.98), myricetin (*R*_f_ ~ 0.75), hyperoside (*R*_f_ ~ 0.55), and rutoside (*R*_f_ ~ 0.38). Regarding phenolic acids, caffeic acid (*R*_f_ ~ 0.93), and chlorogenic acid (*R*_f_ ~ 0.47), were used as chemical standards.

As can be seen in Figure 6, no compounds were separated during the mobile phase's elution for the dichloromethane fraction, and all compounds extracted in this fraction ran completely with the polar mobile phase used, as expected. The mobile phase choice proved to be effective in the separation of the compounds extracted in the ethyl acetate and *n*‐butanol fractions.

Apart from the leaves *n*‐butanol fraction, the remaining fractions indicated many similar compounds for each part of the plant and their analyzed fractions, especially regarding the compounds in higher concentrations. The yellow band with *R*_f_ ~ 0.65 is present in all organs in both ethyl acetate and *n*‐butanol; this band does not match with any of the standards' substances applied on the plate; however, it does match with the pale orange on the same *R*_f_, from an ethanolic decoction prepared in a previous year (batch 2023‐06‐27). Another band that is present in all samples, even in the fraction of the leaves, is the yellow band with *R*_f_ ~ 0.38, in different concentrations; the color and the *R*_f_ match the rutoside standard, suggesting the compounds in this band might be rutoside or a rutoside derivative. Below and above the main yellow band, also common to all samples, except for leaves butanol fraction, two yellow compounds are present in *R*_f_ ~ 0.60 and in *R*_f_ ~ 0.74, which are also not a match with any of the standard bands. The last most prominent band, present in all samples, in different concentrations, is the yellow band in *R*_f_ ~ 0.94.

As it was mentioned in the paragraph above, the band with *R*_f_ ~ 0.38 might be rutin, as previous authors have reported the presence of this flavonoid in *L. nummularia* 's extracts (Yasukawa et al. 1990; Tóth et al. 2014). Alongside rutin, a richness of other flavonoids and phenolic compounds has been detected in *L. nummularia* (Korta 1970; Yasukawa et al. 1990; Marr et al. 1998; Podolak and Strzałka 2008; Tóth et al. 2012, 2014; Podolak, Koczurkiewicz, Galanty, and Michalik 2013; Podolak, Koczurkiewicz, Michalik, et al. 2013; Deng et al. 2015; Hanganu et al. 2016; Özgen et al. 2020; Weissenstein 2021; Suçiu, Stoicescu, Lupu, et al. 2023; Suçiu, Stoicescu, Lupu, Popescu, et al. 2023); some compounds, more specifically, a triterpene saponin, have shown cytotoxic activity against prostate tumor cell lineages, without affecting normal cells (Podolak, Koczurkiewicz, Michalik, et al. 2013); this stimulates further studies on the chemical composition and on the biological activities of *L. nummularia* .

## 4 Conclusions

This paper provides a detailed description of the morpho‐anatomical characteristics of *Lysimachia nummularia* L., cultivated in Switzerland. It also provides HPTLC screening for flavonoids of different fractions obtained from infusions and ethanol decoction. Its morphology presents characteristics such as simple, opposite, membranaceous and oval to rounded leaves. The roots are rounded and present a central medulla. The stem, rounded with four ribs at the ends, possesses an endoderm, which is consistent with the Primulaceae family. The amphistomatic leaves have glandular trichomes present only in the abaxial epidermis. The mesophyll is dorsiventral. The midrib and petiole are concave–convex in shape and have glandular trichomes. These trichomes are also present on both surfaces of the petals and in the filament. The research also identified the presence of several chemical compounds, such as lipophilic compounds (in cuticles, secretory cavities and endoderm), proteins (in epidermis and phloem) and starch (in the cortex, medulla and mesophyll). Phenolic compounds were found in several parts of the plant, while lignin was detected only in the xylem. The absence of calcium oxalate crystals and alkaloids is an important trait for the differentiation of this species, reinforcing the importance of plant chemistry in taxonomy, while the HPTLC strongly stimulates further studies on the chemical composition of the plant.

## Author Contributions

**Carolina Sabedotti:** conceptualization, investigation, writing – original draft, methodology, writing – review and editing, formal analysis, data curation, validation. **João Vitor da Costa Batista:** investigation, methodology, writing – review and editing. **Bettina Leonhard:** investigation, methodology, writing – review and editing. **Konrad Urech:** conceptualization, funding acquisition, supervision, writing – review and editing. **Mahsa Ahmadinezhad:** methodology, writing – review and editing. **Jakob Maier:** conceptualization, funding acquisition, investigation, project administration, resources, writing – review and editing, software. **Stephan Baumgartner:** conceptualization, resources, supervision, project administration, writing – review and editing. **Fabio Boylan:** investigation, methodology, supervision, project administration, writing – review and editing. **Carla Holandino:** conceptualization, investigation, funding acquisition, writing – review and editing, methodology, project administration, supervision, resources. **Jane Manfron:** conceptualization, investigation, writing – original draft, methodology, visualization, writing – review and editing, project administration, supervision, data curation.

## Funding

This work was supported by the Coordenação de Aperfeiçoamento de Pessoal de Nível Superior (88887.974351/2024‐00) and Verein für Krebsforschung.

## Disclosure

Correspondence and requests for materials should be addressed to C.S., S.B., C.H. or J.M.

## Conflicts of Interest

The authors declare no conflicts of interest.

## Data Availability

The data that support the findings of this study are available from the corresponding author upon reasonable request.
